# Complete mitochondrial genome of the isabelline wheatear *Oenanthe isabellina* (Passeriformes, Muscicapidae)

**DOI:** 10.1080/23802359.2016.1167641

**Published:** 2016-06-20

**Authors:** Shaobin Li, An Luo, Guopan Li, Wei Li

**Affiliations:** a College of Life Science, Yangtze University, Jingzhou, China;; b Institute of Biomedicine, Yangtze University, Jingzhou, China

**Keywords:** Mitochondrial genome, passerine, *Oenanthe isabellina*, Tibet Plateau

## Abstract

The isabelline wheatear *Oenanthe isabellina* was widespread in Northern Hemisphere, but the information of this species is poorly known. In this study, the complete sequence of *O. isabellina* mitochondrial genome was obtained by L-PCR and conserved primer-walking approaches. The results showed that the entire mitochondrial genome of *O. isabellina* was 16,812 bp in length with 52.7% A + T content; the genome harbored the same gene order as that of other birds, including 2 rRNA genes, 22 tRNA genes, 13 protein-coding genes and 1 non-coding control region (D-loop). All protein-coding genes of *O. isabellina* mitochondrial genome started with an ATG codon, except for COI with GTG. For terminate codon usage, most of the genes use TAA or TAG. The control region of *O. isabellina* was located between tRNA-Glu and tRNA-Phe with 1244 bp length, no repetitive sequence. The mitochondrial data are potentially important for understanding this poorly known species.

The isabelline wheatear (*Oenanthe isabellina*) is a migratory and insectivorous bird species. The birds have a wide distribution, breeding across the Northern Hemisphere and wintering in Africa and India (Cramp 1988; Zheng 2002). Its altitudinal ranges are from 1000 m up to more than 3400 m *a.s.l*. (del Hoyo et al. 2005). Various open habitats are occupied by them, such as steppes, heaths, shrubland and tundra (Cramp 1988). As a secondary cavity-nesting species, they often build its nest in unoccupied burrows of other species (Li & Lu 2012). It is one of the Eurasian passerines breeding highest on mountains and furthest North. The wheatear is the representative species of its genus to have adapted to these harsh high-altitude conditions. Unfortunately, the life history of isabelline wheatears is poorly understood, except for some simple descriptions mentioned above.

The genus *Oenanthe* was formerly thought to belong to the family Turdidae, but it is now more generally considered to be an Old World flycatcher (Muscicapidae) according to two mtDNA genes (Zheng 2002; Outlaw et al. 2010). Blast results of the entire mitochondrial genome in GenBank also suggest that it is a closer relative of *Luscinia cyanura* (91% sequence similarity, Figure 1) and its relatives in Muscicapidae than *Turdus hortulorum* (87% sequence similarity) with their relatives of Turdidae.

**Figure 1.**
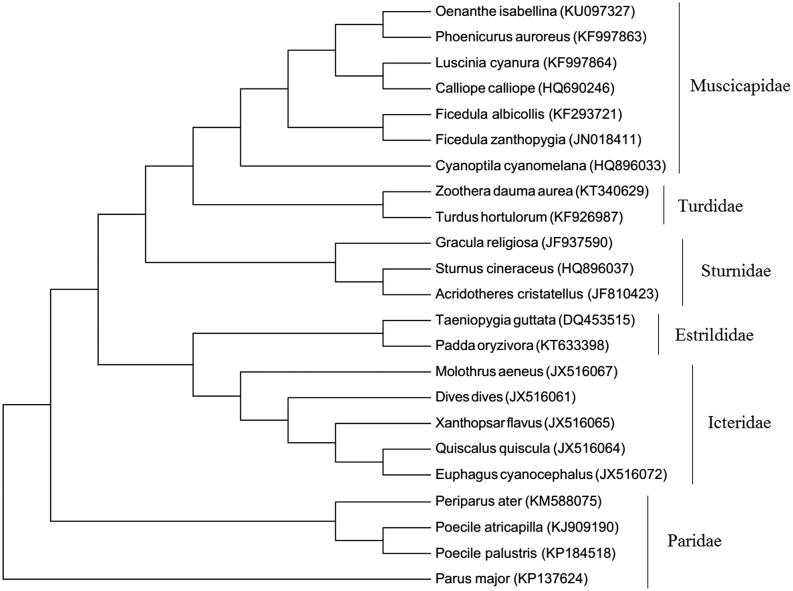
Maximum parsimony phylogenetic trees for 23 related avian species using their complete mitochondrial genome with the accession number in parentheses.

In this study, we sequenced the entire mitochondrial genome of the isabelline wheatear to provide basic data for molecular phylogenetics and further studies on this poorly known species. Blood sample of the isabelline wheatear was collected by puncturing the brachial vein from an individual caught in mist net, on 16 July 2015 at Tianjun County of Qinghai Province (37°17' N, 99° 00' E; 3430 m *a.s.l*.). The sample is stored in the specimen room of College of Life Science at Yangtze University (accession number: IW-2015-2001). The complete sequence of *O. isabellina* mitochondrial genome was determined using L-PCR and conserved primer-walking approaches. The results showed that the entire mitochondrial genome of *O. isabellina* was 16,812 bp in length and has been deposited in GenBank with accession number KU097327.

The nucleotide is composed of 30.0% for A, 14.3% for G, 22.7% for T and 33.0% for C. The A + T content is 52.7%, which is similar to another sympatric ground-dwelling passerine *Pseudopodoces humilis* (52.5%; Xin et al. 2015). It exhibited the typical mitochondrial genome structure of birds, including 2 rRNA genes, 13 protein-coding genes, 22 tRNA genes and a non-coding control region (Figure 1[AQ]). All protein-coding genes of the *O. isabellina* mitochondrial genome started with an ATG codon, except for COI with GTG. Most of the genes terminated with codons TAA or TAG, ND1 and ND5 terminated with AGA, COII with AGG, and the COIII and ND4 genes had an incomplete termination codon (T––). The control region of *O. isabellina* was located between tRNA-Glu and tRNA-Phe with 1244 bp length.
